# Association Between Diet And Cognitive Performance In Adults Aged 60 And Over: NHANES, 2011–2014

**DOI:** 10.1093/geroni/igab046.2641

**Published:** 2021-12-17

**Authors:** Md Towfiqul Alam, Elizabeth Vasquez, Sandra Echeverria

**Affiliations:** 1 University of North Carolina at Greensboro, Greensboro, North Carolina, United States; 2 University of Albany, University of Albany, New York, United States; 3 University of North Carolina at Greensboro, University of North Carolina at Greensboro, North Carolina, United States

## Abstract

There is limited evidence examining associations between diet and cognitive performance (CP) in older adults. We used the 2011-2014 National Health and Nutrition Examination Survey to determine if meeting dietary recommendations was associated with CP in adults 60+ years of age. Diet was based on the healthy eating index (HEI) 2015 and categorized into quintiles (higher quintiles indicating healthier diet). CP was based on word list learning, animal naming, and digit symbol substitution test, with scores above 25th percentile indicating adequate performance. Multivariate logistic regression modeling was conducted and adjusted for potential cofounders. A total of 3,068 participants completed the CP tests. A slightly higher percentage of participants were female (54.0%), predominantly White (80.5%) and the largest percentage (54.7%) was 60 to 69 years of age. The mean HEI-2015 score (0-100) was 54.89 (SE = 0.56). High CP scores increased with healthier dietary quintiles. However, results were only significant (p for trend <0.05) for digit symbol substitution test when comparing those in the highest quintile (82.53%) to those in the lowest (70.23%). Compared with participants in the lowest quintile of HEI-2015, participants in the highest quintile had a two-fold increased odds of better digit symbol substitution test scores, after adjusting for confounders (Odds Ratio [OR]: 1.96, 95% Confidence Interval [CI]: 1.28-3.01). Results showed that meeting healthy diet recommendations is associated with improved digit symbol substitution test, a marker of attention, processing speed and executive function. Future research should consider the role of diet in older adults to improve cognitive performance.

